# Micronuclei and Genome Chaos: Changing the System Inheritance

**DOI:** 10.3390/genes10050366

**Published:** 2019-05-13

**Authors:** Christine J. Ye, Zachary Sharpe, Sarah Alemara, Stephanie Mackenzie, Guo Liu, Batoul Abdallah, Steve Horne, Sarah Regan, Henry H. Heng

**Affiliations:** 1 The Division of Hematology/Oncology, Department of Internal Medicine, University of Michigan, Ann Arbor, MI 48109, USA; 2 Center for Molecular Medicine and Genomics, Wayne State University School of Medicine, Detroit, MI 48201, USA; zasharpe@umflint.edu (Z.S.); sarahalemara03@gmail.com (S.A.); smmackenzie@crimson.ua.edu (S.M.); gliu@med.wayne.edu (G.L.); batoulabdallah@gmail.com (B.A.); stevendhorne@gmail.com (S.H.); saregan@bu.edu (S.R.); 3 Department of Pathology, Wayne State University School of Medicine, Detroit, MI 48201, USA

**Keywords:** cancer evolution, chromosome aberrations, chromosomal coding, fuzzy inheritance, genome chaos, genome instability, genome re-organization, micronuclei, micronuclei cluster, non-clonal chromosome aberrations or NCCAs, system inheritance

## Abstract

Micronuclei research has regained its popularity due to the realization that genome chaos, a rapid and massive genome re-organization under stress, represents a major common mechanism for punctuated cancer evolution. The molecular link between micronuclei and chromothripsis (one subtype of genome chaos which has a selection advantage due to the limited local scales of chromosome re-organization), has recently become a hot topic, especially since the link between micronuclei and immune activation has been identified. Many diverse molecular mechanisms have been illustrated to explain the causative relationship between micronuclei and genome chaos. However, the newly revealed complexity also causes confusion regarding the common mechanisms of micronuclei and their impact on genomic systems. To make sense of these diverse and even conflicting observations, the genome theory is applied in order to explain a stress mediated common mechanism of the generation of micronuclei and their contribution to somatic evolution by altering the original set of information and system inheritance in which cellular selection functions. To achieve this goal, a history and a current new trend of micronuclei research is briefly reviewed, followed by a review of arising key issues essential in advancing the field, including the re-classification of micronuclei and how to unify diverse molecular characterizations. The mechanistic understanding of micronuclei and their biological function is re-examined based on the genome theory. Specifically, such analyses propose that micronuclei represent an effective way in changing the system inheritance by altering the coding of chromosomes, which belongs to the common evolutionary mechanism of cellular adaptation and its trade-off. Further studies of the role of micronuclei in disease need to be focused on the behavior of the adaptive system rather than specific molecular mechanisms that generate micronuclei. This new model can clarify issues important to stress induced micronuclei and genome instability, the formation and maintenance of genomic information, and cellular evolution essential in many common and complex diseases such as cancer.

## 1. Introduction 

“Micronuclei” refers to the small sized nuclei that form from one or a few chromosomes or fragments of one or a few chromosomes when they are not incorporated into one of the daughter nuclei during cell division [1]. Micronucleus formation usually serves as an index of genotoxic effects and chromosomal instability (both inherited and induced). For example, micronuclei testing represents an important tool for assessing DNA damage, defects in mitosis, and the stress response. It is also used for monitoring an individual’s genome instability and recently for studying immune activation following DNA damage [2,3,4]. 

Various molecular mechanisms can lead to the formation of micronuclei, including double-stranded DNA breaks, impaired DNA repair response, improper DNA replication, direct and indirect results from the treatment of DNA adduct-forming chemicals, crosslinking agents, and interference with mitosis itself often through inhibition of microtubule polymerization or centromere interference. Cancer cells may contain double minutes (one type of supernumerary marker chromosomes that are characterized by a typical dumbbell shape, representing extra-chromosomal materials resulted from gene amplification), extrachromosomal DNA containing amplified genes, that have been linked to interphase formation of nuclear buds, micronuclei, and tumorigenicity [5]. Micronuclei are also linked to various chromosome aberrations such as defective mitotic figures (DMF), chromosome fragmentations (C-Frags), mitotic cell death, mitotic catastrophe, giant nuclear, and especially genome chaos [6,7,8,9,10,11,12,13,14] (see Table 1).

**Figure 1:**
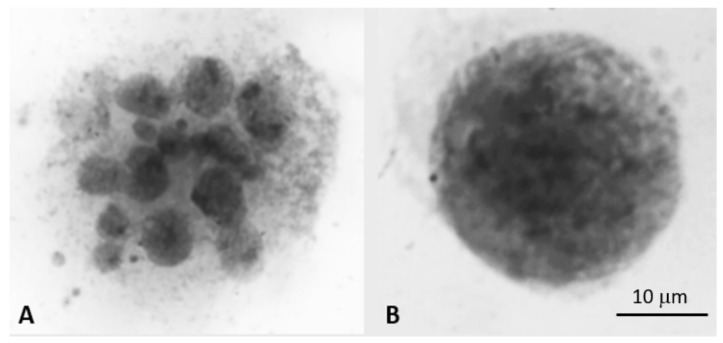
Example of the micronuclei cluster. **A**) represents a micronuclei cluster (with a dozen of smaller nuclei) induced from Doxorubicin (2 μg/mL, for 2 h) treated mouse ovarian surface epithelial (Brca1 -/- and p53-/-) cells. For the treatment details please see Liu et al., 2014 [11]. **B**) represents a normal size nucleus from the control group. Both images are reversed DAPI (4′,6-diamidino-2-phenylindole, a fluorescent DNA stain) images. The high frequencies of micronuclei clusters can be induced from different drugs or treatments including inhibitor of the Aurora B kinase, and various irradiations.

**Figure 2:**
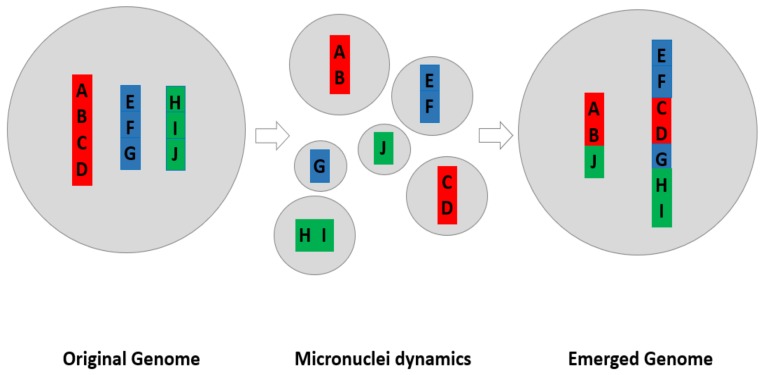
The diagram of how micronuclei create a new genome by re-organizing karyotype coding. When under stress (either internal or environmental), the cluster of micronuclei is formed, which can either lead to death, proportional survival (partial population survival without altering the genome), form an emergent genome through fusion/fission cycle, or simply combine with other nuclei.

Micronuclei have clearly been observed in many cell types under many different conditions (See Figure 1). However, what is the key biological function along with being used as an index of genome instability? Micronuclei have been shown as a mechanism of elimination of genetic material, such as amplified MYCN genes from neuroblastoma cells (The MYCN gene is a cellular proto-oncogene of the MYC family of transcription factors) [7]. Recently, they have been linked to fuzzy inheritance as their evolutionary potential has increased [13,14], which led to the realization that these often-observed micronuclei clusters from cancer cell populations are of somatic evolutionary significance (See Figure 2). Based on the recent realizations that genome instability is a common driver in cancer evolution and that micronuclei belong to a type of non-clonal chromosome aberrations (NCCAs) [18,21,22,24,25], increasing interests are now focusing on the biological significance of micronuclei. With the recent linkage of micronuclei and nuclear envelope dynamics (e.g., mis-segregated chromosomes recruiting their own nuclear envelope (NE) to form micronuclei) and genome chaos such as chromothripsis [26,27], and particularly the association with immune activation [28], it is easily anticipated that micronuclei studies will soon attract more molecular researchers. 

However, based on our experience of studying complex bio-systems, when new factors are identified (e.g., nuclear envelope and immune activation) and associated with specific molecular features (e.g., micronuclei), the complexity of such features often drastically increase when more researchers with diverse backgrounds rush into the field. Paradoxically, the increased complexity usually decreases the value of these identified factors after extensive studies on them, as many factors are usually involved in certain bio-events and it would be challenging to identify the dominating molecular mechanism among many others. This situation has been illustrated by various studies, including the oncogene [13,14], aneuploidy [22], ER (endoplasmic reticulum) stress [29]. This was the rationale for us in developing the evolutionary mechanism of cancer over large numbers of diverse individual molecular mechanisms [24,30,31]. In fact, such phenomena can be applied to most complex systems when emergent properties are involved and when there are many amounts and types of agents at the lower level [13,32].

While studying specific molecular mechanisms of micronuclei is of importance, merely focusing on individual molecular mechanisms will obviously not suffice in understanding the system complexity, especially when a specific mechanism is only one among many and is not the dominating causative factor [12,13,33]. The strategy should be switched to studying the first principle and identifying common mechanisms using the lens of evolutionary and complex systems [13,22]. To illustrate how to achieve this goal, in this commentary we will briefly review the history and current issues of micronuclei research and discuss a few examples of ways to search for a common cause, universal key function, and the general consequences of micronuclei. Such analyses aim to provide a fundamental understanding of micronuclei and its relationship with genome instability and evolutionary potential, which is the basis for many common and complex diseases. 

## 2. Brief History of Micronuclei Research

Micronuclei (MN) were first discovered in erythrocytes by Howell and Jolly, leading to the term Howell-Jolly bodies [34]. Micronuclei research was fairly stagnant until their formation was observed in root tips of kidney beans after neutron radiation exposure, providing a link between DNA damage and micronuclei formation [35]. The establishment of this correlation has led to the use of the micronucleus as a marker of DNA damage and this has dominated the field. A micronucleus test for assessing DNA damage in bone marrow samples was developed and proved to be easier than the standard analysis of using metaphase chromosome spreads to look for chromosome fragments [36]. This protocol also set forth the criteria for counting micronuclei, a micronucleus being 1/20th to 1/5th the size of the main nucleus [36].

The micronucleus test was further improved by Countryman and Heddle via the use of peripheral lymphocytes instead of bone marrow samples, making the test easier to perform on samples taking from human subjects [37]. The lymphocytes were exposed to ionizing radiation and then stimulated with the mitogen phytohemagglutinin (PHA); micronucleus formation was then observed having a dose-dependent relationship to radiation exposure [37]. This dose-dependent response provided a further basis for the micronucleus test as an assay for potential genotoxins, as concentrations of chemicals or other stresses such as radiation dosage could now be deemed unsafe. Countryman and Heddle also developed a technique for small-scale culture of human peripheral blood lymphocytes, reducing the amount of sample needed and further improving efficiency [38].

Countryman and Heddle also developed an inclusion criteria for micronuclei to reduce false positives. A micronucleus was defined as being 1/3 or smaller compared to the main nucleus, with a similar or slightly lighter staining intensity. There could be no overlap or contact of the micronucleus with the main nucleus and it had to be non-refractile to rule out dye stain. Finally, the micronucleus had to be within 3 to 4 nuclear diameters of the main nucleus and no more than two micronuclei could be associated with the same nucleus [37].

The micronuclei test still had several shortcomings. Foremost, a tissue sample may have different numbers of actively dividing cells, and therefore raw numbers of micronuclei between two samples may not be comparable [39]. This issue was solved by the development of the cytokinesis-block micronucleus (CBMN) assay. Under this protocol, samples were treated with cytochalasin B, which inhibits contraction of the cleavage furrow actin microfilament during cytokinesis, resulting in binucleate cells that have completed nuclear mitosis but are not successfully separated [40]. Under this condition, only binucleate cells (those which are shown to be actively dividing) are scored for micronucleus formation, and micronucleus frequency is expressed as a ratio of micronuclei per binucleated cell, rectifying the previous problem [40]. Importantly, Fenech et al. 1997 shows that scoring undivided cells only for micronuclei may greatly underestimate chromosomal instability, as undivided cells showed greatly decreased numbers of micronuclei compared to binucleated blocked cells when subjected to identical genotoxic stresses [41].

While this scoring was useful for in vitro sample testing to rule out damage that may have occurred when the tissue was subjected to the experimental stress, it was pointed out that this method would greatly underestimate chromosomal instability or CIN from lymphocytes taken in vivo. It was recommended that in vivo samples be scored twice: 24 h after PHA exposure and after cytokinesis block, including micronuclei found in mononucleated cells in both counts [42].

Furthermore, protocols for culturing lymphocytes and criteria for scoring micronuclei along with reporting frequencies differed greatly between investigating labs, making large-scale comparisons and drawing conclusions from data challenging. The HUman MicroNucleus (HUMN) collaboration began in 1997 to standardize the use of the CBMN assay in investigating and reporting micronucleus formation frequencies. A set of criteria for scoring micronuclei was developed and published to labs for use in CBMN analysis of PBLs based on the Fenech criteria [43]. A later survey by HUMN displayed how participating labs conformed to the accepted criteria, with the clear majority of surveyed labs conforming to the Fenech/HUMN criteria [44].

The modern micronucleus assay uses the CBMN protocol but also scores other aberrations such as nucleoplasmic bridges (NPBs), nucleoplasmic buds (NBUDS), necrotic, and apoptotic cells; this serves as an assessment of the “cytome” or overall chromosomal stability of a cell population [45]. This development is useful because extreme CIN may result in apoptosis or other cell death [16].

Today, several uses for the micronucleus test exist. The CBMN assay is now a mainstay of genotoxicity testing, where a potential genotoxin can be applied to cells in vitro or to an animal model in vivo and PBLs drawn and tested for micronuclei formation. Micronucleus testing has been used to investigate the effects of nutritional supplementation on chromosomal instability. For example, folate deficiency was correlated with increased micronuclei formation [46]. This could have important implications for cancer prevention and treatment. It has been proposed as a way to rapidly screen large numbers of people for radiation exposure while integrating new technology [47]. Such advances increase the public health and disaster response utility of micronucleus testing and study.

## 3. Examples of Linking Micronuclei to Biological Functions and Cancer

Since micronuclei belong to one type of NCCA [8,12,13,14] and the frequency of NCCAs represents an index of CIN [7,18,19], it is logical to study micronuclei in cancer, as CIN is the common driver of cancer [8,19,21,24]. The overlapping mechanisms for both CIN and micronuclei were previously used as a rationale to study their involvements in cancer. For example, CIN is often discussed with chromosome segregation in mitosis from spindle or centromere defects, the same problems that leads to micronucleus formation. Therefore, micronuclei are but one expression of CIN [48].

Many micronuclei studies in cancer can be grouped into different categories based on the nature of their studies (either mechanistic or application studies of micronuclei as clinical markers for anti-cancer therapy via a variety of approaches, as a marker for estimating cancer risk, or simply to measure the level of CIN). The following are some examples:

- Link between micronuclei and cancer risk: Micronuclei are much more easily quantifiable than other types of chromosomal aberrations and the protocol and scoring of the CBMN assay is much easier than that of assessing metaphase chromosomes. A strong link has developed between chromosomal instability and cancer, with micronucleus formation being indicative of CIN. The current consensus is that micronuclei are linked to cancer risk, although there is a fair amount of variability in predictive power among cancer types [49]. The HUMN collaborative proved a useful tool to develop a clinical test based on micronuclei using their previously published criteria for scoring micronuclei in peripheral lymphocytes. Through analysis of data on micronucleus frequency studies in cancer patients and controls, there was found to be a strong link between urogenital and colon cancer development and patients who had micronuclei frequencies defined as medium to high [49]. Additionally, increased micronucleus frequency in lymphocytes has had variable correlations with lung and pancreatic cancer risks that may become validated with further studies [50,51]. Micronuclei are also being evaluated in cervical cells as a possible result of HPV infection and link to cervical cancer development [52]. Epithelial cells, due to the ease of access, have long been an attractive target for such a clinical test, but due to the common nuclear anomalies seen in these cell types that could have been mistaken for micronuclei, new methods had to be developed [53]. This work has laid the basis for further investigation of epithelial cell micronuclei. The HUMN collaborative has worked on a validation and coordination process for using buccal epithelial cells in the CBMN-Cyt protocol to develop an even easier method to access tissue as a clinical test for cancer risk [54]. A comparison of the developing technique between three laboratories showed a 20-fold increase in MN frequency among cancer patients compared to controls [55].
- Tumor radiosensitivity: Tumor cells have been exposed to differing doses of radiation in vitro; the more micronuclei per binucleate cell after cytokinesis blocking, the less the surviving fraction of cells [56]. These results imply that micronucleus formation is correlated with cell death, a point corroborated by other studies that linked micronuclei formation during mitosis to eventual apoptosis [57]. There have been issues drawing conclusions about the micronuclei link to radiosensitivity in vivo.
- Post-therapy assessment of tumors: Increased chromosomal instability has generally been associated with progression and poor outcome [58] but the link between increased micronuclei formation and apoptosis has opened up some therapeutic possibilities, resulting in a paradox: CIN has been viewed as beneficial in some studies and harmful in others. Is there an explanation? Some studies show that an increase in already rather unstable tumor cells may “tip the balance” toward apoptosis, resulting in tumor reduction [59,60]. This highlights the importance of being able to measure CIN.
- Elimination of double minutes: The amplified genes represented by double minutes have been shown to be able to be removed from the cell by micronucleus formation [61,62]. As many elements contributing to invasiveness and poor outcome are carried in double minutes, this represents a possible therapeutic goal. Radiation therapy at low levels has been shown to expedite the removal of double minutes via micronucleus formation [63].
- Relationship with genome chaos: Micronucleus formation may be a driver of CIN and chromosomal damage, not just a consequence of it. Micronuclei frequencies have been shown to increase in cells that have undergone checkpoint adaptation, entering mitosis with damaged DNA, a process normally prevented via the p53 pathway [64]. When the p53 pathway has been inactivated, numerous structural aneuploidies including micronuclei are able to form [65]. The resulting micronuclei may contain DNA that becomes damaged further via nuclear envelope collapse [66]. This damaged DNA may then be reintegrated into the genome, resulting in a localized chromosome rearrangement known as chromothripsis, a common feature of many cancers [9,67,68]. Some early studies have in fact shown that not all micronuclei can simply be eliminated, but can perform biological functions such as DNA synthesis and rejoining into other nuclei and further contributing to abnormal karyotypes [69,70]. However, like most NCCAs, they were regarded by the research community as insignificant background noise and have largely been ignored [7,71] Recently, time lapse microscopy has confirmed the fact that many micronuclei are actively involving the CIN process. However, without the theoretical framework to explain the meaning of altered karyotypes and how MN essentially contribute to new karyotypes, such observations would have failed to illustrate the importance of the micronuclei in cancer. The discovery of the association between punctuated microcellular evolution and karyotype chaos or genome chaos highlighted the importance of studying various unclassified types of chromosomal and nuclear abnormalities, including micronuclei [11,12]. The later realization that increased genome alterations are essential for cellular adaptation also put forth a positive perspective to this idea [12,13,14,29]. It was also realized that the altered karyotype represents a new genomic information package, as the order of genes along chromosomes represent genomic coding [23,72]. Thus, like many other types of NCCAs, the micronuclei are not just the products of biological errors or “bad” outcomes, but are also important components of the dynamics of the systems essential for cellular adaptation [13,14,21,22]. Recently, micronuclei have been linked to various genome chaos-related chromosomal and nuclear abnormalities, including “budding/bursting” and horizontal transfer [73,74,75,76]. Despite their different mechanisms of induction, they share the same evolutionary mechanism of creating new genomic information (more see later discussions). In fact, it has long been known that micronuclei have been detected in cells from patients with chromosomal instability/cancer susceptibility syndromes (e.g., Ataxia telangiectasia and Bloom syndrome) [77,78]. It is also known that elevated CIN can be detected from these patients. The linkage between CIN and micronuclei is obvious.
- Link between immuno-systems: Recent studies have established that micronuclei activate the innate immune response [2,3,4,28]. Specifically, exposing fragmented DNA to the cytoplasm triggers the activation of IFN-I and IFN-stimulated inflammatory genes, which suggest the presence of micronuclei can be sensed by the cell defense machinery. Similarly, it was shown that double-stranded DNA breaks lead to the formation of micronuclei, which precede the activation of inflammatory signaling and are a repository for the pattern-recognition receptor cyclic GMP–AMP synthase. Such links between DNA damage, micronuclei, and immunity offer a new avenue to studying cancer evolution and drug treatment response.
- Some key features of micronuclei differ from primary nuclei: Interestingly, not just the size difference, but both the degree of chromatin condensation and nuclear envelope composition differ between micronuclei and primary nuclei. Micronuclei also lack active proteasomes [26]. Together, the relationship between micronuclei and primary nuclei provides an excellent example of the emergent relationship between parts vs. entire system, as such emergence is not just about the quantitative difference, but different systems.

## 4. New Challenges and Opportunities for Micronuclei Research

With increased molecular characterization of the interactions between micronuclei and CIN and micronuclei with immunoactivity, the research landscape is rapidly shifting. It can be anticipated that this cytogenetic field will soon attract many more molecular investigators who are keen on pathway analyses. While it is exciting, there are some important issues that need to be addressed. Specifically, the recently proposed genome theory needs to be introduced as a new conceptual framework which is essential for the success of this re-emergent field.

### 4.1. Re-Organizing the Genome: A New Genomic and Evolutionary Framework for Understanding the Function of Micronuclei

#### 4.1.1. The Recently Realized Genomic Information Context: System Inheritance and Fuzzy Inheritance

To fully understand how micronuclei contribute to the creation of new genomic information, one first needs to appreciate the concept of system inheritance. For decades, genetic information has been mainly limited to the gene coded “parts inheritance” (and recently the epigenetic information has received more attention), but the more important genomic information, which functions as the information context of individual genes, has been largely ignored. “System inheritance” is essentially defined by the topological arrangement of the genes, regulation elements, and other DNA elements along and among chromosome [12,13,14,23,72]. Such higher-level genomic information encodes how gene interactions occur and represent a new coding system. By accepting the concept of chromosomal coding, the micronuclei’s function becomes obvious as it always involves the alteration of chromosomes, and thus the genomic information. Like other non-clonal chromosome aberrations, micronuclei contribute to the fuzzy inheritance by increasing the level of genome heterogeneity involved. [21,22].

**Figure 3:**
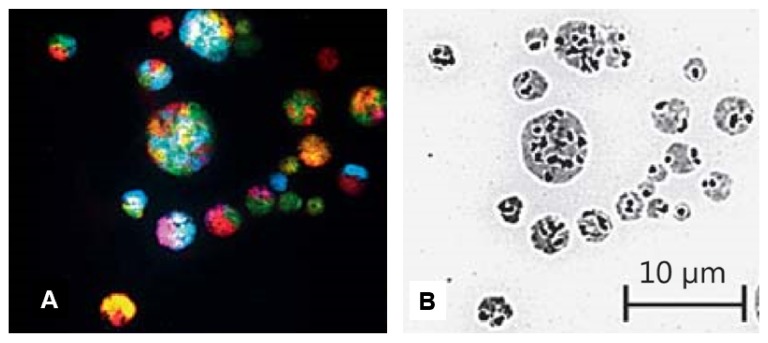
Spectral karyotyping (SKY) image of a micronuclei cluster. Panel A is the SKY (spectral karyotyping) image of a micronuclear cluster. Different colors represent different chromosomes. While the two biggest micronuclei contain numerous chromosomes, most of the smallest micronuclei only contain single chromosome (indicated by one color). Panel B is the same image with reversed DAPI staining. The strong black dot signals represent the centromere (suggesting that all nuclei of this cluster contain a centromere). This micronuclear cluster was observed from re-cultured cell population of mouse ovarian surface epithelial (Brca1 -/- and p53-/-) cells. Following the treatment of Doxorubicin (2 μg/mL, for 2 h). Figure 3 is reused from reference [12], with permission from Karger.

Although many micronuclei display similar morphology, they do have different genomic contexts or display different system inheritance. Due to their small size, most micronuclei do not have sufficient chromosomal material to form a functional genome. The chromosomal content of micronuclei cluster in cancer cells have been analyzed using SKY (Spectral karyotyping), for example, and different micronuclei clearly contain different chromosomes, judged by the pure and mixed colors (in SKY testing, each chromosome is labeled as one unique color) [11,21] (also see Figure 3). Therefore, micronuclei themselves and the cellular population displaying the high level of micronuclei are different systems when compared to normal cellular populations, as they do not have an original karyotype.

There are two different strategies used to study the mechanism of micronuclei. One can focus on the specific molecular pathways that are associated with micronuclei (either the pathways leading to micronuclei or events/phenotypes directly resulting from micronuclei). Such studies need to follow the process of micronuclei. Many future molecular characterizations (including gene mutations) likely belong to this category. For example, the detailed molecular pathways that lead to micronuclei, how micronuclei trigger immuno-response, and how micronuclei lead to chaotic genomes.

Another strategy is to focus on the cells that are generated from the fusion or fission of MN. Since either the generation of micronuclei, or fusion of micronuclei to form viable (almost normal sized) nuclei, will involve genomic information changes, the generation of micronuclei becomes an effective means to producing the genomic variants essential for cellular adaptation and/or macroevolution. Paying less attention to specific types of molecular mechanisms, the key should be to focus on the process’ impact on the surviving cell populations rather than micronuclei themselves. As discussed in a later section, it is the end product, which is the surviving cell population, that really matters.

#### 4.1.2. Evolutionary Context: Stress Induced Genomic Variants and Its Evolutionary Significance Coupled with Trade-Off

In the past few decades, cancer research has generated a large number of diverse individual molecular mechanisms [12,30,31,79]. In addition to many of them conflicting with each other, some of them were clearly able to be both “good” and “bad” for cancer depending on the context of the environmental and evolutionary stages of the cellular populations. For example, the same gene can function as an oncogene or tumor suppressor and the same mechanism (such as aneuploidy) can either inhibit or promote cell proliferation of a given population. To unify these individual mechanisms, the concept of the evolutionary mechanism of cancer was proposed based on stress induced genome variations and genome based evolutionary selection [20,24,30,31]. Since most individual pathways, when under stress, can lead to genome variations and can be favored by cancer evolution, they can all be linked to cancer under experimental conditions but are often not the common causative factor for cancer in a patient’s population. The mechanism of cancer and its relationship with large numbers of molecular factors explains why most molecular mechanisms of cancer have failed to be applied in clinical practice. The same rationale has been used to explain how aneuploidy [22] and different types of NCCAs contribute to cancer [12,13]. Since the environments are highly dynamic and any variant has the ability to be both good or bad depending on the context, the prediction factor based on specific molecular pathways is low. Such uncertainly also can be found in various experimental systems where the off-target effect and artificial effect can be highly significant [80]. Transfection methodologies may have off-target effects, even when an empty vector is used as an intended negative control; the stresses placed on the cells during transfection experiments can have a cytotoxic effect and the cells that survive may be irrevocably different in their behavior [80]. This may make drawing reproducible conclusions difficult.

Under such frameworks, the stress condition can induce both micronuclei and cells with altered genomes (genomic variants). In addition, the fusion-fission cycle can effectively produce cells with new genomic contexts through the process of genome chaos (Figure 2) [7,10,12,13]. Despite massive cell death, some surviving cells (with new genomes) were able to be selected by macroevolution. Thus, macroevolutionary selection is based on the cell population with new karyotypes rather than micronuclei, as most of them will be eliminated (even though continuously produced when the genome is less stable). Following the success of a macroevolutionary phase, some cancer gene mutations can promote an increase in the population size of cancer cells through the microevolutionary phase.

#### 4.1.3. Limitation of Specific Pathways Used to Explain a Highly Complex System Behavior: Lower Level Agents vs. Higher Level Emergence

Like most complex biological systems, the phenotype of a higher-level system is due to the emergence of lower level agents, and often with no one-to-one correlation between system behavior and their lower-level agents [13,21,25]. The same rule can be applied to the MN’s function. Thus, the prediction is mainly based on when parts specificity is low. Even transitional MNs can be a participating factor in the emerging phenotype, which makes the predicting ability low due to too many involved variants (both parts and the dynamic process).

Like other adaptive biological processes, the dynamics of micronuclei display some common features: Macroevolution (e.g., speciation) is often not driven by gene mutation, thus does not often depend on fixed specific pathways or events; it is not the micronuclei themselves, but the cell population with the altered genome that is the target of evolution. If even micronuclei can activate the immune-system, the new genomes should contribute more to the activation of the immune-system. The processes of genome-micronuclei and new genome processes is linear and prediction based on pathways is insignificant.

Considering all three points, the mechanistic study of micronuclei and its consequences should be through the lens of an adaptive system where system inheritance and cellular evolution play a key role. Despite the interests of each individual mechanism, the research focus at research communities should not be heavily focused on them due to the fact that there are so many, and due to the frequent pathway switching. In contrast, monitoring the overall evolutionary potential is a better strategy.

Therefore, the most important synthesis of the biological meaning of micronuclei dynamics is the evolutionary selection based on the creation of new genomic information packages. The meaning of these stochastic combinations of micronuclei is the increased new genomic information at cellular population level, rather than individual specificity. No matter which mechanism leads to these micronuclei, they all belong to the different agents that may or may not contribute to the emergence.

#### 4.1.4. The Model of How Micronuclei Contribute to New Genome Systems by Creating New Genomic Coding

Known consequences of micronuclei include (1) generation of chromothripsis under experimental conditions; they are linked to drug induced genome chaos; they are associated with nuclear budding and giant cells (under experimental conditions, the giant cells can form many micronuclear cluster); they are directly linked to aneuploidy, (2) they can change the chromosomal compositions and introduce translocated chromosomes (e.g., aneuploidy can trigger translocations), and (3) they can involve highly diverse molecular mechanisms and despite their difference, a fundamental hidden linkage is that they all contribute to create new genome systems by genome re-organization.

Figure 2 illustrates this model of how micronuclei contribute to new genome formation.

As illustrated in Figure 1, the generation of micronuclei cluster can often be observed from cancer cell lines, especially when these cell lines are unstable and under stress. These MN clusters often involve fusion and fission cycles. It has been proposed that such fusion/fission cycles represent trial-and-error in searching for viable genomes [12,13,21]. The micronuclei can clearly promote this process. This model of different mechanisms to promote the formation of new genome systems by reorganizing new karyotypes is clearly applicable in explaining micronuclei dynamics. It can also explain why there is a correlation between increased micronuclei and with drug resistance, metastasis, and other macro-cellular evolution related phase transitions [12]. In these cases, the common genomic mechanism is increased fuzzy inheritance at the genome level, ultimately leading to new system inheritance.

It should be mentioned that this model is similar to the model of genome chaos generating a new re-organized genome under stress, a survival strategy for cancer cell populations [10,31]. The key is rapid and massive changes at the genomic context by changing karyotypes. In a sense, the involvement of micronuclei is less chaotic.

As for micronuclei formation without the involvement of micronuclei cluster and the fusion-fission cycle, it still changes the karyotypes for some cells, which changes the degree of genome heterogeneity, although often less drastic.

### 4.2. The Need for Further Research to Clarify Important Issues

#### 4.2.1. Differentiating Micronuclei Generated from Relatively Stable Genomes and Micronuclei Clusters from Unstable Genomes

Traditionally, issues regarding the morphological features and frequencies of micronuclei have been straight forward. The vast majority of micronuclei study has focused on the frequency of micronuclei formation, primarily as a rate per dividing cells. However, many published criteria sets that may exclude important information are restrictive. The observation of multiple mitotic figures in cancer for example, suggests that chromosomal instability may be underestimated if these cells are not included in micronucleus frequency counts.

Beyond frequency, size of micronuclei represents another important feature. There is a strong correlation between the size of the micronucleus and its centromere presence, with centromere-positive micronuclei being larger than centromere-negative micronuclei. This can allow for differentiation between primarily aneugenic and clastogenic stress [81]. Size-based quantification can have similar accuracy in determining centromere presence to that of FISH (fluorescence in situ hybridization) using centromere probes [82]. It is important to note that while performing micronuclei assessment after genotoxin exposure in vitro can determine the nature of the genotoxin, centromere assessment on cells obtained from a patient may lead to less conclusive results as micronuclei formation in vivo is the cumulative sum of countless cellular stresses. Additional information regarding the size of micronuclei and the method used to score them is listed in Table 2.

Little data has been published regarding micronucleus shape and morphology; most published criteria states that a micronucleus must be round and smooth in order to be counted. However, research with some mechanisms (such as ionizing radiation) has been seen to produce irregularly shaped micronuclei [83].

Now with the increased study of using NCCAs (including micronuclei) to monitor cancer evolution [13,14,19,24] by linking the micronuclei to genome chaos, both the frequencies and morphological heterogeneity have become important issues deserving immediate attention.

While induced frequencies of micronuclei from cancer samples or cell lines can be very high, the size and shape are highly dynamic (Figure 1 and Figure 3), which significantly differ from traditional micronuclei described in Table 2.

Shockingly, the cluster of micronuclei can involve active fusion and fission despite cell death ([21] Ye et al., unpublished observations). Since for those small nuclei there are only limited chromosomal materials (Figure 3), it makes sense that the fusion process is essential in forming viable nuclei which have sufficient chromosomal materials. As illustrated in the model (Figure 2), the key biological function of fusion/fission among micronuclei cluster is to change the genomic information for the generation of new emergent genomes. Despite that the majority of MNs will be eliminated, even a tiny portion of them can change the game of evolution. This is the reason the genome chaos process is so important in cancer.

Now knowing the difference (from morphology to biological function) between micronuclei cluster and the traditional micronuclei, a key question is whether or not these different types of mechanisms are involved in producing them? It is known that the base level of CIN is very different, for example. It would be interesting to compare the cell death pattern with the pattern of new genome emergence.

#### 4.2.2. Awareness to Confusing Issues

To obtain the ideal platform for micronuclei data interpretation, the classification of micronuclei is needed prior to scoring them. For example, research is needed to compare the types of micronuclei and their relative contribution to CIN, as micronuclei can be classified into micronuclei with or without a centromere (likely resulting from genotoxic stress); classical micronuclei or micronuclei clusters; and micronuclei in cell lines with high or low levels of genome instability.

In the literatures of micronuclei studies, there have been some terminologies/concepts regarding micronuclei that need clarification.

a. Micronuclei and cell death:

Although most micronuclei will be eliminated, many of them will clearly involve the new genome formation. The ignorance towards altered non-clonal chromosomal aberrations in the past was also influenced by the viewpoint of the cell death process. It was thought that as soon as the apoptotic pathway was activated, targeted cells and micronuclei were eliminated, which resulted in the attitude of “why bother”. The experiment of tracing cancer evolution in action however proved otherwise [8,17,19,24]. As discussed in the subject of cell death heterogeneity [16], cell death can not only happen in an incomplete form but can also contribute to fast and rapid evolution by forming new genomes!

The same consequences apply to micronuclei. First, not all of micronuclei are eliminated. Second, even when many of the micronuclei are eliminated, they still contribute to the alteration of genomic information; furthermore, many micronuclei are constantly eliminated and generated and presented at a given degree of fuzzy inheritance; the cell death process paradoxically produces genome chaos to allow new cell populations to emerge, which displays new genotypes and phenotypes such as drug resistance. Systematic studies of how maximal cell death therapy could lead to rapid drug resistance through genome chaos clearly illustrates this point ([13,14] Horne et al., unpublished data).

Interestingly, the reversible cell death process has received much attention. Various types of cell death, including mitotic cell death [9,17,84], apoptosis [85,86], necroptosis—a programmed version of necrosis [87], and entosis [88] can all reverse their own process. The majority of research is currently focused on cell death pathways. Significantly, by linking the overall dynamic changes of the transcriptome, survival was pinpointed by the new genome rather than specific pathways [16,89]. A large number of pathways are also identified for reversible apoptosis, including activation of pro-cell survival, anti-oxidation, cell cycle arrest, histone modification, DNA-damage, and stress-inducible responses, and at delayed times, angiogenesis and cell migration [86] Based on the observations that a proportion of survived cells (generated from the reversible cell death process) display chromosomal abnormalities and other genetic defects, which is associated with malignant growth, it is most likely that they share the same evolutionary mechanism of genome chaos. With a genome-based framework, it becomes much easier to understand the phenomena of reverse cell death. It is not the reversal of a single cell’s death, but a behavior of the cell population after some of their members emerge with new genomes [13,14]. It is thus useful to study whether or not micronuclei also contribute to the reversible cell death process. Even entosis, despite its bizarre behavior, has a similar evolutionary mechanism of micronuclei fusion: changing the genome coding by integrating new genomic materials and creating new information packages.

b. Fragmentations of DNA, chromosomes and nuclei:

Since micronuclei are associated with DNA fragmentation (DNA damage), chromosome fragmentation (mitotic cell death), and nuclear fragmentation (burst dividing), it is necessary to distinguish them and to develop methods to characterize them. Even for chromosome fragmentation there are different subtypes [90].

To describe the phenomena of chromosome fragmentation, different terms including chromosome pulverization and premature chromosome condensation (PCC) are used. With similar morphologies, chromosome pulverization and PCC have been previously studied with a certain degree of confusion. Some studies for example have confused chromosome pulverization with PCC. Following the identification of chromosome fragmentation, the similarities and differences between chromosome fragmentation and PCC were compared [90] and there are many differences between them.

c. Micronuclei can be linked to genome chaos (including chromothripsis), as well as many other stress related factors.

Despite the observation that chromothripsis has been experimentally induced with micronuclei [91], we should not consider micronuclei to be the main causative factor for chromothripsis. In contrast, the high level of stress represents a major and common factor which can trigger genome chaos (both locally, reflected as chromothripsis; and the entire genome, reflected as a massive chaotic genome) [11,15]. Based on the cytogenetic analysis of experimentally induced genome chaos, chromothripsis accounts for 10% of all induced chaotic genomes. It is likely that this proportion would be much higher if the DNA sequence of chaotic genomes were compared based on a single cell profile. The reason that less massive chromosomal re-organizations are detected from patients’ samples is due to the fact that sequencing methods are not able to profile the non-clonal chromosomal aberrations. In addition, most massive chaotic genomes are transitional types, which play an important role in phase transition but are less detectable in the end products of evolution [12,13,14,24].

Again, for all subtypes of genome chaos, including chromothripsis, the main influence is the re-organized coding system. Future studies of micronuclei and cancer evolution should focus on this general evolutionary mechanism.

Moreover, the relationship between micronuclei and different types of NCCAs in other human diseases need to be studies, as NCCAs are clearly linked to different diseases [92,93,94]. Equally important, how micronuclei play a role in the somatic mosaicism need more attention. In recently years, the issue of somatic mosaicism has received increased attentions [95,96,97].

Abnormal karyotypes have been linked to a higher chance of poor outcome in acute myeloid leukemia patients, which supports the use of karyotyping as a diagnostic tool [12,13,14,15]. The genome theory is able to explain these findings as a high level of CIN along with karyotypic heterogeneity between tumor cells, which allows the previously mentioned genome “reshuffling” to find a new workable system. Consistent cytogenetic analysis may allow the evaluation of a tumor’s capability to rapidly adapt to stress and should be broadened by the inclusion of NCCAs such as micronuclei [15,21,22]. As chromosomal instability, and thus the capability of the genome system to change when stresses are applied can be measured by NCCAs, it is important that previously ignored chromosomal abnormalities and NCCAs are investigated further. Evaluation of karyotypic heterogeneity increases in importance when genotoxic drugs are used for chemotherapy, as these may force tumor cells to “adapt or die”, resulting in most of the original tumor being killed but also driving the generation of a variety of new genome systems that may form a new cancer [93,94]. The ultimate expression of karyotypic heterogeneity comes in the form of somatic chromosomal mosaicism, in which a significant portion of an individual’s cells display varied karyotypes; this has the potential to provide a higher baseline variability and may predispose these individuals to disease [95,96,97]. A deeper study of such individuals and their predisposition to complex diseases such as cancer may help to develop ways to evaluate the link between karyotypic heterogeneity and disease progression [13,14].

## 5. Conclusions

By briefly reviewing the field of micronuclei research, and particularly by discussing some important differences between classic micronuclei (detected from cells with relatively stable genomes) and micronuclei clusters (detected from cells with unstable genomes), our manuscript has emphasized the ultimate importance of studying micronuclei through the lens of complexity and genome mediated evolution. To understand the dynamic relationship between micronuclei (as well as other genomic variants) and human diseases, the key is to appreciate the karyotype defined genomic information and its changes by responding to various stresses. The model of micronuclei contributing to new genome systems is thus of importance and can help build the future of micronuclei research.

In regards to some of the mentioned issues above, the relationship between MNs and other chromosomal aberrations should be investigated. Particularly, how to quantitatively measure NCCAs and how to deal with the outliers among the average. Just like other NCCAs, the increased heterogeneity of micronuclei can increase the evolutionary potential, which can function to either improve cellular adaptation or increase the odds for diseases as a trade-off [21,22,29].

Finally, the entire process of generating micronuclei and ending up with a new genome is highly dynamic, with which so many individual molecular mechanisms can be involved. Focusing on system behavior at a higher level, is much more useful than characterizing each of these many individual pathways. In other words, simply focusing on creating a long list of individual molecular mechanisms is of no use, a lesson still to be learned in current cancer research. As pointed out by Dr. Robert Weinberg, over half a century of cancer research has generated an enormous body of molecular data, unfortunately, “…there were essentially no insights into how the disease begins and progresses to its life-threatening conclusions” [73]. Clearly, micronuclei research should avoid the same incompetent methods as studying evolutionary mechanism is ultimately important [13,14,21,22,74].

## Figures and Tables

**Table 1:** Explanations of key concepts/terminologies.

**Genome Chaos**	a. Definition: Genome chaos, a process of complex, rapid genome re-organization, results in the formation of chaotic genomes, some of which are selected to establish stable genomes. It was initially detected by cytogenetic analyses, and recently confirmed by whole-genome sequencing efforts, which identified multiple subtypes including “chromothripsis”, “chromoplexy”, “chromoanasynthesis”, “chromoanagenesis”, and “structural mutations” [11,13]. b. Mechanism: A diverse set of molecular mechanisms representing various stress conditions can trigger genome chaos. Genome chaos acts as an evolutionary mechanism by providing a survival strategy under stress, when the re-organization of the genome creates new genomic coding, the system inheritance. During this process, non-homologous end joining (NHEJ) is likely involved [11,13]. c. Biological significance: Genome chaos represents an effective means for both cellular adaptation and organismal speciation. It plays an important role in macro-cellular evolution and possibly is responsible for a rapid speciation event during massive extinction [14].
**The relationship between genome chaos and other types of chromosomal or nuclear abnormalities**	a. Chromothripsis is a subtype of chaotic genome: Despite the current popularity of chromothripsis, it belongs to one of many different types of chaotic genome. Due to the limited scale of genome re-organization, chromothripsis can be favorably selected by evolution and detected by sequencing methods. In contrast, when multiple chromosomes are involved, these very complicated chromosomal changes often exist in a non-clonal form (e.g., non-clonal chromosome aberrations (NCCAs)), thus are often un-detected by current sequencing methods. According to the in vitro model, chromothripsis makes up roughly less than 10 percent of all different types of chaotic genomes examined [11,13,15]. b. The process of genome chaos can unify mitotic catastrophe (previously loosely defined cell death resulted from aberrant mitosis or failed segregation. The Nomenclature Committee on Cell Death has suggested the use of other terms such as “cell death at metaphase” or “cell death preceded by mutinucleation”), chromosomal fragmentation (mitotic cell death associated with massive chromosomal elimination) [9,16,17], and many other abnormalities including giant nuclei [10], micronuclei cluster, and entosis. Some important perspectives of genome chaos unify these abnormal nuclear structures/behaviors. First, they are associated with stress mediated microcellular evolution. During the induction of genome chaos, including different types of cellular transition, elevated nuclear abnormalities of all types can be detected. Second, they all have been considered as a dead end without further biological significance. In fact, that was the main reason researchers have ignored all these structures for a long time. It was thus very surprising when it was realized that the uncompleted cell death can contribute to cancer evolution [8,9,18,19]. Third, and perhaps most importantly, despite all morphological differences, all types of chromosomal or nuclear abnormalities (or variants to be correct) provide altered system inheritance, directly or indirectly, thus that evolutionary selection can work on them.
**The relationship between stress and genome alteration-mediated evolution**	a. Stress response is the key evolutionary mechanism (both short term adaptation and punctuated macroevolution during crisis) [20]. Importantly, a highly diverse set of molecular mechanisms can be unified by stress-mediated genomic variants. The high stress phase of macro-evolution has been observed from human and mouse in vitro transformation models [8,18,19], as well as drug resistant models. Interestingly, genome chaos has been observed in many normal tissues, suggesting that even moderate levels of stress can trigger genome re-organization. As for the types of stresses, most stress types examined thus far can induce genome chaos, as long as they can lead to cell death [14,18,19]. b. The level of stress, the frequencies of outliers, and the phase of evolution are key contributors for the emergence of new systems. Even though the level of stress is a obvious dominating factor, other factors such as inherited genome instability and the phase of evolution have an impact on the frequencies of genome chaos. Note that in the in vitro model to induce genome chaos, the dosage of drug is within the range of the clinically used dosage. More importantly, genome chaos can be produced by the somatic cell evolutionary process without any drug treatment. Clearly, the elevated genome alterations function as both materials and the results of cellular evolution. At the earlier stage of studies, people often link the elevated genome chaos to a specific drug/dosage and cell line. Now, the elevated genome alterations have been observed under many stressful conditions. c. Macro-cellular evolution: The game of outliers. Even though it is a rather complicated issue to predict somatic evolution, the frequencies of genome chaos and the overall population instability serves as a better measure of prediction. For lower levels of stress, as long as the system is highly unstable, the frequencies of altered genomes coupled with system instability will allow some outliers to become winners. This feature is drastically different from in normal physiological conditions where the average (normal cells or clonal populations) can overpower outliers [13]. It should point out that, for cell populations that display high level of CIN (chromosomal instability), many drugs can induce the genome chaos at the rate of over 90% to 100%.
**Fuzzy inheritance:** Understanding the diverse morphological variations of chromosomes and nuclei	It has been very confusing why there are many stochastic chromosomal variants. Traditionally, it was thought that these non-clonal abnormal structures were insignificant “noise”. They also were considered as the results of bio-errors. Following mapping of the non-clonal chromosomal aberrations into the dynamic phase of cancer evolution, it was realized that all these non-clonal variants represent evolutionary potential. Furthermore, the search for biological meaning of these highly diverse genome level variants has finally led to the concept of fuzzy inheritance [13,14,21,22]. Based on a new understanding of the genomic basis of bio-heterogeneity, it is now realized that the inheritance itself needs to be heterogenous as well. In contrast to the gene theory, which states that a gene codes for a specific, fixed phenotype, the genome theory suggests that most genes code for a range of potential phenotypes depending on context provided by other genes and the environment. From this “fuzzy” range of phenotypes, the respective environment can then allow the best-suited status to be “chosen” [22]. Such inheritance that codes for a range of phenotypes, not just a fixed phenotype, is named as fuzzy inheritance. Fuzzy inheritance can be observed at the gene, epigenetic and genome level. Furthermore, genome instability can increase the fuzziness of the inheritance.Equally important, the fuzzy inheritance explains why there are so many diverse nuclear variations including all types of micronuclei, especially under high stress or within the phase of macro-evolution. It is now clear that despite different morphological features and different mechanisms to produce them (either directly from near 2n cell or giant cells with >4n), these fundamentally represent fuzzy inheritance at the somatic cell level, with the evolutionary function remaining the same, changing the genome encoded genomic information.
**Genome Theory**	Departing from gene theory where genes determine the individual’s characteristics, and represent the independent informational unit, genome theory considers that the genomic topology serves as the context of the gene interactions, and genomic inheritance is about the network structure determined by chromosomal coding (gene order along and among chromosomes). There are 12 principles for genome theory. Among them, that the genome organizes the interactive relationship among genes, the genome is the main platform for evolutionary selection, and the genome functions as a main constraint for a given species are some of the important ones [13,14,23].

**Table 2:** Comparison of Scoring Criteria for the Micronuclei Test. Several inclusion criteria for counting micronuclei to determine frequencies during a micronucleus test, primarily in genotoxicity analysis. While these criteria are effective in reducing false positives and work well for the CBMN-Cyt protocol in validated tissue types, they may not be universally applicable to all cell types (especially with the high variance found in cancer cells). Additionally, many traits beyond micronucleus frequency have not been thoroughly investigated, especially in regard to usefulness as a biomarker of chromosomal instability.

Characteristic	HUMN Standardization Criteria: Peripheral Blood Lymphocytes [43]	Tolbert 1992 Exfoliated Cells [53]	Heddle 1976 Peripheral Blood Lymphocytes [37]	HUMN Laboratory Survey [44]	Comments
Size	1/16 to 1/3 of diameter of main nucleus	Less than 1/3 of diameter of main nucleus; no lower limit if shape and color are discernable	Less than 1/3 of main nucleus	1/16 to 1/3 of diameter of main nucleus (91% of labs surveyed)	Multinucleated cell division and fusion as seen in single cancer cells and genome chaos may alter these criteria, as several nuclei much larger than the average micronucleus may result.
Shape	Round or oval with own membrane	Round and smooth, suggests a membrane	N/A	Round or oval (99%) Morphologically identical to main nucleus (62%)	Micronuclei may have abnormal shapes depending on mechanism and integrity of the nuclear envelope. A disordered/disrupted nuclear envelope in a micronucleus-type structure likely leads to DNA content loss but is still a reflection of chromosomal instability.
Stain	Same staining intensity as main nuclei, but can be slightly darker	Mostly same as main nuclei	Mostly same as main nuclei or lighter	Same intensity as main nucleus (83%) Same color as main nucleus (85%)	Micronuclei may be on a separate “condensation schedule” than the main nucleus which may contribute to DNA damage to their genetic content
Overlap/Contact	May not overlap with main nucleus but can touch itMust have a distinguishable boundary with no bridge, not linked or connected	No overlap or bridge with main nuclei	No overlap or contact with main nuclei	Overlapping with or touching main nuclei is allowed as long as it is distinguishable (49%) No bridge to main nuclei (73%)	HUMN regards micronucleus-like structures continuous with the main nuclear membrane as nuclear buds (NBUDS); these are often formed in interphase from double minutes. Inclusion of NBUDs in scoring systems often treats them as a separate phenomenon.
Other	Non-refractile	Same focal plane as main nuclei Feulgen-positive	Non-refractile Within 3 to 4 nuclear diameters of main nuclei No more than 2 micronuclei associated with same nucleus	Non-refractile (95%)	
